# Construction of a Label-Free Electrochemical Immunosensor Based on Zn-Co-S/Graphene Nanocomposites for Carbohydrate Antigen 19-9 Detection

**DOI:** 10.3390/nano11061475

**Published:** 2021-06-02

**Authors:** Chia-Wei Su, Jia-Hao Tian, Jin-Jia Ye, Han-Wei Chang, Yu-Chen Tsai

**Affiliations:** 1 Department of Chemical Engineering, National Chung Hsing University, Taichung 402, Taiwan; a70137@smail.nchu.edu.tw (C.-W.S.); zpxoci1950@gms.tku.edu.tw (J.-H.T.); asd7721609@gmail.com (J.-J.Y.); 2 Department of Chemical Engineering, National United University, Miaoli 36001, Taiwan

**Keywords:** Zn-Co-S/graphene nanocomposites, electrochemical immunosensor, carbohydrate antigen 19-9

## Abstract

Nanocomposites of the binary transition metal sulfide Zn-Co-S/graphene (Zn-Co-S@G) were synthesized through a one-step hydrothermal method. They may be useful in the construction of an electrochemical immunosensor for carbohydrate antigen 19-9 (CA19-9) detection. Zn-Co-S dot-like nanoparticles uniformly covered the surface of graphene to form an interconnected conductive network, ensuring strong interaction between transition metal sulfide and graphene, which can expose numerous electroactive sites leading to the improvement of the amplified electrochemical signal toward a direct reduction of H_2_O_2_. Thus, the construction of an electrochemical immunosensor using Zn-Co-S@G nanocomposites showed outstanding sensing properties for detecting CA19-9. The constructed electrochemical immunosensor exhibited a good linear relationship in the range of 6.3 U·mL^−1^–300 U·mL^−1^, with the limit of detection at 0.82 U·mL^−1^, which makes it a promising candidate for an electrochemical immunosensor.

## 1. Introduction

High specificity, reliability, and sensitivity of tumor biomarker sensing platforms are expected to play a significant supporting role in enabling personalized medicine and point-of-care testing for future drug development and clinical cancer diagnostics. Biomarkers, such as carbohydrate antigen 19-9 (CA19-9), consisting of the macromolecule glycoprotein exist in tumors, and have been shown to be strongly associated with malignant tumors (mainly pancreatic, colorectal, liver, gastric, and ovarian). Typically, the CA19-9 concentrations are lower than 37 U·mL^−1^ for normal healthy people; a slight increase of CA19-9 concentration may cause a significant increase in the relative risk of cancer incidence and development [1]. Over recent decades, many research groups have developed reliable methods for CA19-9 biomarker sensing platforms, including enzyme-linked immunosorbent assays (ELISA) [2], surface-enhanced Raman scattering spectroscopy (SERS) [3], chemiluminescence [4], electrochemiluminescence [5], and electrochemical immunosensors [6], with a positive influence on diagnostic values of CA19-9 for early cancer diagnosis and surgical prediction. Among them, electrochemical immunosensor consisting of biological molecules and electrochemical active nanocomposites garnered considerable attention owing to their advantages of simple operation procedure, cost-effective production, and high sensitivity. Currently, electrochemical immunosensors can mainly be classified into non-labeled (label-free) and labeled (sandwich-type) immunosensors. In contrast to the non-labeled form, labeled immunosensors often require time-consuming, complicated, and expensive processes to cause an increase in the denaturation of biomarkers that lead to unfavorable effectiveness. For this reason, it is of great significance to develop a label-free electrochemical immunosensor to provide remarkable biomarker sensing. The key to developing label-free electrochemical immunosensors is to create a sensing interface composed of electrochemically active sites and good electrically conducting substrates. It has been demonstrated that the fabrication of electrochemical sensors using transition metal sulfide nanomaterials could possess excellent catalytic ability originating from their multiple oxidation states and unique hierarchical structure. Hence, they are ideally suited to the use of transition metal sulfide nanomaterials as a potential electrode material for label-free electrochemical immunosensors. Recent investigations have shown that transition metal sulfide nanomaterials, such as MoS_2_ [7], CoS_2_ [8], CuS [9], and NiCo_2_S_4_ [10], were suitable for preparing electrochemical immunosensors that displayed the superior sensing performance. Such an effect was acknowledged to be partly caused by the lower electronegativity of S, which exhibited remarkable electrochemical performance [11,12]. Considering the attractive properties of graphene, greatly enhanced electrocatalytic sensing can be achieved by integrating graphene with transition metal sulfide nanomaterials. The unique 2D graphene nanostructure with plenty of binding sites and excellent biocompatibility provides an excellent platform to support the growth of transition metal sulfide nanomaterials to form transition metal sulfide/graphene nanocomposites and tune the electrocatalytic sensing properties. Kubendhiran and Wang et al. revealed that the existence of unique 2D graphene nanostructures could exhibit remarkable electrochemical catalytic activity because graphene materials could expose more edge sites that lead to faster growth of transition metal sulfide materials to form transition metal sulfide/graphene hybrids and further deliver better electrochemical performance in biomarker detection [13,14]. The proposed transition metal sulfide/graphene nanocomposites have higher electrode/electrolyte contact areas to provide more electroactive sites and effectively facilitate the electronic/ionic transport kinetics. This could be built upon to gain a great deal of attention in the development of electrochemical immunosensors for the identification of additional cancer-specific tumor biomarkers and provide preferred approaches for early diagnosis and further prognosis guidance. In this work, the binary transition metal sulfide Zn-Co-S/graphene nanocomposites were synthesized through a one-step hydrothermal method. A graphene supported by Zn-Co-S dot-like nanoparticles serving as electroactive sites for the electrochemical reactions is a potentially promising candidate for future electrochemical immunosensors.

## 2. Materials and Methods

### 2.1. Reagents

Zinc nitrate hexahydrate (Zn(NO_3_)_2_·6H_2_O), cobalt nitrate hexahydrate (Co(NO_3_)_2_·6H_2_O), thioacetamide (TAA), and natural graphite flakes were obtained from Alfa Aesar (Ward Hill, MA, USA). Chitosan and bovine serum albumin (BSA) were purchased from Sigma-Aldrich (St. Louis, MO, USA). Carbohydrate antigen 19-9 (CA19-9) and CA19-9 antibody (anti-CA19-9) were purchased from BioSource (Camarillo, CA, USA). All water used was deionized water (DI water) through a Milli-Q water purification system (Millipore, Billerica, MA, USA). All chemicals were used without further purification.

### 2.2. Preparation of Zn-Co-S/Graphene Nanocomposites

Graphene oxide (GO) was synthesized from natural graphite flakes by using a modified hummers method [15]. The Zn-Co-S/graphene nanocomposites were prepared via a one-step hydrothermal method. As is typical, 0.0765 g of Zn(NO_3_)_2_·6H_2_O, 0.1464 g of Co(NO_3_)_2_·6H_2_O, 0.1840 g of TAA, and 60 mg of GO were dissolved in 40 mL of DI water under continuous stirring for 30 min until the mixture dissolved completely. This was followed by ultrasonic treatment (for 1 h) to achieve a homogeneous aqueous solution. The homogeneous aqueous solution was transferred to a 100 mL Teflon-lined stainless steel autoclave and then heated to 190 °C for 6 h. After this, the autoclave was cooled to room temperature and the resulting Zn-Co-S/graphene nanocomposites (denoted as Zn-Co-S@G) were separated by centrifugation, washed with ethanol and DI water three times, and dried in an oven at 60 °C before being collected for subsequent treatment.

### 2.3. Fabrication of Electrochemical Immunosensor 

**Scheme 1:**
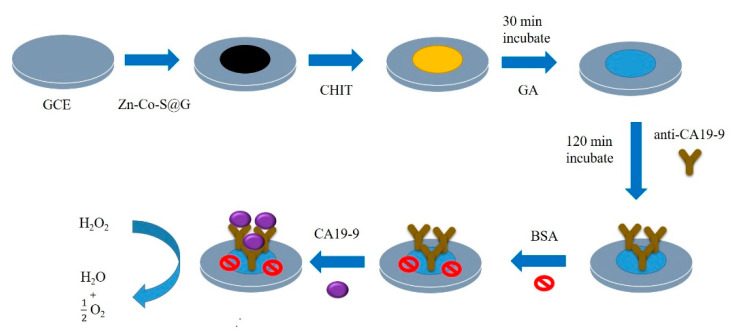
The fabrication of the electrochemical immunosensor.

The fabrication of the electrochemical immunosensor as shown in Scheme 1. A total of 2 mg of Zn-Co-S/graphene nanocomposite was mixed in 1 mL of ethanol by ultrasonication for 10 min to get a well dispersed solution, then 6 μL dispersed solution was dropped on the surface of a glassy carbon electrode (GCE) to prepare the Zn-Co-S@G/GCE. Chitosan (CHIT) (50 mg) was dissolved in 10 mL of 1% aqueous acetic acid to prepare the chitosan solution and drop-casted on the surface of the Zn-Co-S@G/GCE to form CHIT/Zn-Co-S@G/GCE and then completely dried. The formed CHIT/Zn-Co-S@G/GCE was immersed into a 2.5% glutaraldehyde (GA) solution for 30 min to produce imine bonds (C=N), forming GA/CHIT/Zn-Co-S@G/GCE, and then washed with phosphate buffered saline (PBS) to remove unbound GA. Subsequently, the substrate was immersed into an 80 μg·mL^−1^ solution of anti-CA19-9 for 150 min, which was used for the C=N bond-forming processes with GA, forming anti-CA19-9/GA/CHIT/Zn-Co-S@G/GCE, and washed with PBS to remove unbound antibodies. Finally, the substrate was incubated in bovine serum albumin (BSA) solution (0.25%), to block the unreacted active sites on the electrode surface, and then washed with PBS. The electrochemical immunosensor was achieved, denoted BSA/anti-CA19-9/GA/CHIT/Zn-Co-S@G/GCE, and could be used directly as an electrochemical immunosensor and incubated in CA19-9 solution to complete the immunoreaction.

### 2.4. Apparatus

The morphology was characterized by using field emission scanning electron microscopy (FESEM, JSM-7410F, JEOL, Tokyo, Japan) and field emission transmission electron microscopy (FETEM, JEM-2100F, JEOL, Tokyo, Japan). The chemical structure and composition were determined by X-ray photoelectron spectroscopy (XPS, PHI-5000 Versaprobe, ULVAC-PHI, Kanagawa, Japan). X-ray diffraction (XRD) was recorded using a D8 Discover (Bruker, Darmstadt, Germany) X-ray diffractometer with Cu Kα radiation. Electrochemical measurements were performed using a three-electrode system by an electrochemical analyzer (Autolab, model PGSTAT30, Eco Chemie, Utrecht, Netherlands). The electrochemical cell was comprised of a prepared sample-modified GCE electrode, a platinum wire counter electrode, and an Ag/AgCl (3 M KCl) reference electrode in 0.1 M PBS (pH = 7.4) in the absence and presence of hydrogen peroxide (H_2_O_2_). Error bars represent the standard deviations for the 4-times repeated measurements. Electrochemical impedance spectroscopy (EIS) was obtained at frequencies from 0.01 Hz to 100 kHz with an AC perturbation of 5 mV in 0.1 M PBS (pH = 7.4) containing 5 mM [Fe(CN)_6_]^3−^ as an electrochemical probe.

## 3. Results

**Figure 1:**
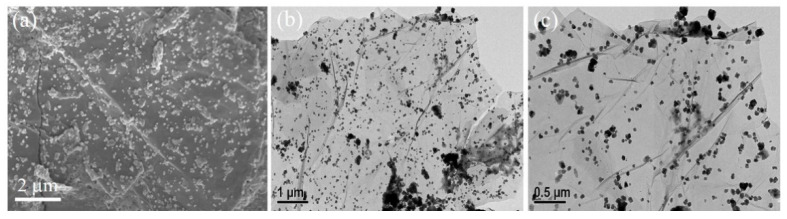
(**a**) FESEM and, (**b**,**c**) FETEM images of Zn-Co-S@G nanocomposites.

The morphologies of Zn-Co-S@G nanocomposites were characterized by FESEM and FETEM. The FESEM image (Figure 1a) reveals that the Zn-Co-S grew into dot-like nanoparticles on the surface of graphene sheets and that the average size was found to be around 50–100 nm. As expected, similar morphology was also clearly seen in the FETEM images (Figure 1b,c). FESEM and FETEM observations identified that Zn-Co-S dot-like nanoparticles uniformly cover the whole surface of graphene to form an interconnected conductive network. Such unique structural characteristics ensure a large effective surface area and provide high electroactive regions and short diffusion lengths, illustrating that the excellent conductivity of Zn-Co-S@G nanocomposites can facilitate the fast transport of electrons and electrolyte ions within the electrode material to effectively promote its electrochemical activity. It could, therefore, be seen as a promising candidate for the fabrication of electrochemical immunosensors.

**Figure 2:**
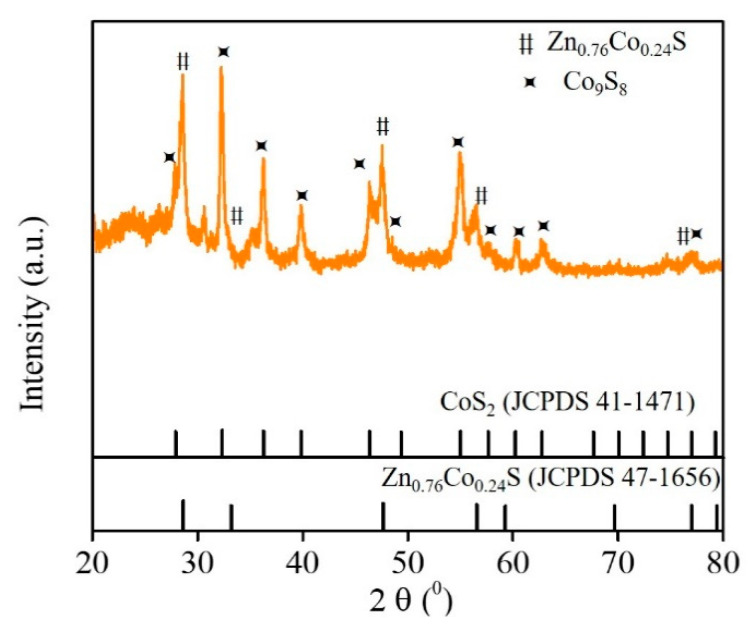
XRD patterns of Zn-Co-S@G nanocomposites.

**Figure 3:**
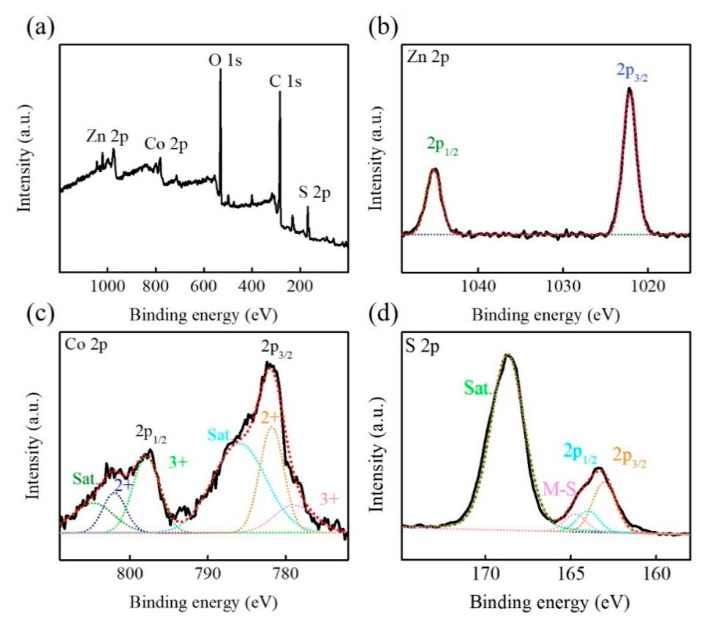
XPS paterns of Zn-Co-S@G nanocomposites (**a**) full scan, (**b**) Zn 2p, (**c**) Co 2p, and (**d**) S 2p.

The phase and surface composition of Zn-Co-S@G nanocomposites were characterized by XRD and XPS. In the XRD results in Figure 2, Zn-Co-S@G nanocomposites exhibit the well-resolved characteristic peaks at 2θ of about 28.6°, 33.1°, 47.7°, 56.5°, and 77.0°, corresponding to (111), (200), (220), (311), and (331) planes of pure cubic phase of Zn_0.76_Co_0.24_S (JCPDS No.47-1656) matching, and at 2θ of about 27.9°, 32.4°, 36.3°, 39.9°, 46.3°, 55.1°, 58.0°, 60.5°, 62.9°, and 77.0°, corresponding to (111), (200), (210), (211), (220), (311), (222), (230), (321), and (331) planes of CoS_2_ (JCPDS No.41-1471) matching [16,17], confirming the ongoing sulfidation process of Zn-Co-S@G nanocomposites. The sulfidation process involved Zn-Co precursor growth on the surface of the graphene, which was subsequently converted to Zn-Co-S sulfide by using thioacetamide (TAA) as a sulfur source. Figure 3 shows the XPS spectra of Zn-Co-S@G nanocomposites to further confirm the fact that Zn-Co-S@G nanocomposites contain mainly the presence of Zn, Co, S, C, and O elements, the chemical and structural characterization of which can be expanded further by the peaks of the XPS spectra. The full scan XPS spectrum (Figure 3a) displays the presence of Zn, Co, S, C, and O elements in the Zn-Co-S@G, suggesting the successful synthesis of the Zn-Co-S@G nanocomposites through the sulfidation process, consistent with the XRD. The Zn 2p XPS spectrum (Figure 3b) shows the spin-orbit splitting of the two peaks located at around 1022.2 eV and 1045.2 eV, corresponding to Zn 2p_3/2_ and Zn 2p_1/2_, respectively. The difference in binding energies between Zn 2p_3/2_ and Zn 2p_1/2_ peaks (about 23.0 eV) confirms that Zn exists in an oxidation state of +2 within Zn-Co-S@G nanocomposites [18]. The Co 2p XPS spectrum (Figure 3c) at 782.0 and 797.4 eV is assigned to Co 2p_3/2_ and Co 2p_1/2_, which are separated by about 15 eV. By fitting Co 2p XPS spectrum with the well-defined shake-up satellites (denoted as Sat.) and the spin-orbit splitting of Co^2+^ and Co^3+^, the results reveal the coexistence of Co^2+^ and Co^3+^ in Zn-Co-S@G nanocomposites [19]. Finally, The S 2p XPS spectrum (Figure 3d) included S 2p_3/2_ and S 2p_1/2_ spin-orbit splitting at 163.0 eV and 164.0 eV with one shake-up satellite (Sat.), demonstrating the presence of S^2−^. Also, compared with the complete peak fitting of the region of S 2p XPS spectrum, it was found that the peak corresponding to metal-sulfur bonds (denoted as M-S) were obtained at 164.8 eV [20]. Overall, the Zn-Co-S@G nanocomposites demonstrated the presence of multiple-valence states of metal, including Zn^2+^, Co^2+^, and Co^3+^, that were counted as potential multiple electrocatalytic active sites, and further provided strong evidence for the promotion of electron transfer between metal and sulfur atoms through the formation of metal-sulfur coordination within Zn-Co-S@G, which ensured strong transition metal-sulfur interactions. The efficient electroactive sites created by involving Zn, Co, and metal-sulfur bonds in Zn-Co-S@G nanocomposites are available for constructing electrochemical immunosensors.

In this study, Zn-Co-S@G nanocomposites were successively fabricated on a glassy carbon electrode (GCE) as a sensitive substrate and applied as a highly-sensitive electrochemical immunosensor. It was expected to provide an effective way to improve electrochemical activity, leading to an amplified electrochemical signal for label-free detection of CA19-9 biomarkers [21]. Small biological molecules such as hydrogen peroxide (H_2_O_2_) produced by inflammatory and vascular cells are well known to induce oxidative stress and contribute to many pathological conditions such as cancer, neurological disorders, and diabetes, etc. Therefore, H_2_O_2_ is commonly exploited as an electroactive species enabling electrochemical sensing, and can provide important aspects of evaluating the electrochemical activity of Zn-Co-S@G nanocomposite [22]. The obtained Zn-Co-S@G nanocomposite immobilized electrode with extended electroactive sites allows the improvement of amplified electrochemical signal toward direct reduction of H_2_O_2_. The mechanism of direct H_2_O_2_ reduction can be illustrated as follows [21,23].

H_2_O_2_ + e^−^ → OH_ad_ + OH^−^ (1)

OH_ad_ + e^−^ → OH^−^ (2)

2OH^−^ + 2H^+^ → 2H_2_O (3)

**Figure 4:**
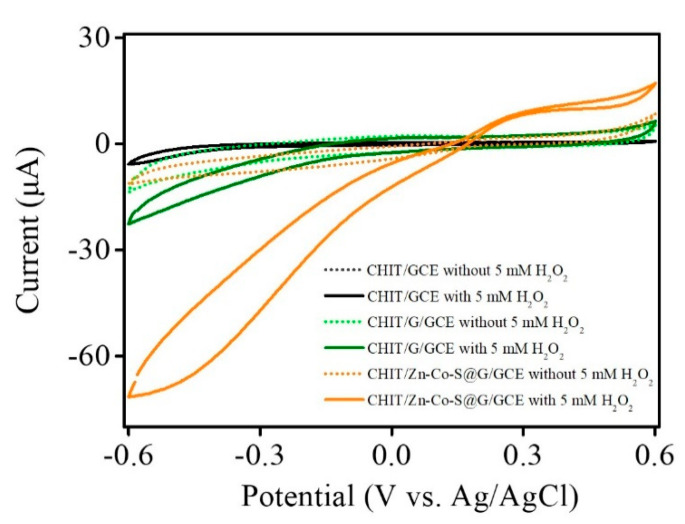
CV curves of CHIT/GCE, CHIT/G/GCE, and CHIT/Zn-Co-S@G/GCE in 0.1 M PBS in the absence (dashed lines) and presence (solid lines) of 5 mM H_2_O_2_ at a scan rate of 50 mV·s^−1^.

Meanwhile, during direct H_2_O_2_ reduction process, the presence of Zn, Co, and graphene in Zn-Co-S@G nanocomposites changes into a more reduced state as electroactive sites facilitate the electron transfer at negative potentials, which may significantly enhance electrocatalytic activity towards direct H_2_O_2_ reduction. Figure 4 shows the cyclic voltammetry (CV) of CHIT/GCE, CHIT/G/GCE, and CHIT/Zn-Co-S@G/GCE in 0.1 M PBS in the absence (dashed lines) and presence (solid lines) of 5 mM H_2_O_2_ at a scan rate of 50 mV·s^−1^. The significant response current after adding 5 mM H_2_O_2_ indicated that the sensing based on these electrode materials exhibited excellent catalytic performance for H_2_O_2_ detection, in addition to H_2_O_2_ reduction. When comparing CHIT/Zn-Co-S@G/GCE with CHIT/GCE and CHIT/G/GCE, it shows a promising sensing property on the H_2_O_2_ reduction response current, which may be due to the synergistic effect between Zn-Co-S and graphene. The positive contribution attributed to the reduction reaction of H_2_O_2_ primarily occurred on multiple electroactive sites in Zn-Co-S@G that are favorable to the construction of electrochemical immunosensors and the improvement of immunosensor performance when detecting the CA19-9 biomarker.

**Figure 5:**
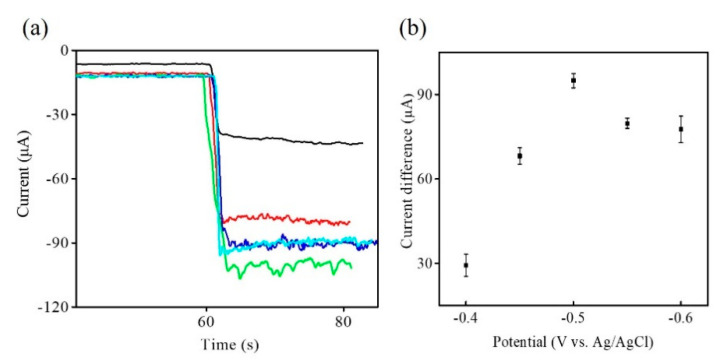
(**a**) Amperometric I-t curve of CHIT/Zn-Co-S@G/GCE with different applied potentials; −0.4 V (black line), −0.45 V (red line), −0.5 V (green line), −0.55 V (blue line), and −0.6 V (cyan line) in 0.1 M PBS in 5 mM H_2_O_2_. (**b**) The relationship between response current and applied potentials in Figure 5a.

To further obtain the best response current to the H_2_O_2_ reduction, the relationship between the response current of CHIT/Zn-Co-S@G/GCE and the applied potentials were optimized by amperometric I-t curve at different potentials (from −0.4 to −0.6 V) in 0.1 M PBS and 5.0 mM H_2_O_2_ (Figure 5a). The changes in the response current of CHIT/Zn-Co-S@G/GCE with applied potentials is presented in Figure 5b. It was observed from Figure 5 that the response current clearly increases with decreasing applied potentials, and reaches a maximum at applying potential −0.5 V. A further decrease applied to the potential results when the response current decreased due to the introduction of surface heterogeneity or instability from adsorbed intermediate products with a low applied potential. The other plausible reason for the selected applied potential of −0.5 V is that the baseline also decreased with a relatively high applied potential (close to −0.5 V), which would also be expected to decrease the reduction-oxidation reactions of the interfering species. So, an applied potential of −0.5 V was chosen as the best applied potential for the subsequent experiments [24,25]. 

**Figure 6:**
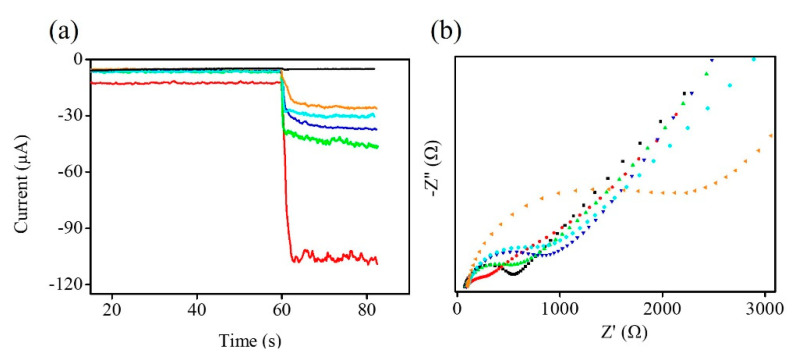
(**a**) Amperometric I-t curve and (**b**) EIS of GCE (black line), CHIT/Zn-Co-S@G/GCE (red line), GA/CHIT/Zn-Co-S@G/GCE (green line), anti-CA19-9/GA/CHIT/Zn-Co-S@G/GCE (blue line), BSA/anti-CA19-9/GA/CHIT/Zn-Co-S@G/GCE (cyan line), and CA19-9/BSA/anti-CA19-9/GA/CHIT/Zn-Co-S@G/GCE (orange line).

During the stepwise electrochemical immunosensor construction process, the electrochemical behaviors of the stepwise modified electrodes were evaluated by the amperometry and EIS. The stepwise modified electrodes were showed by the amperometric I-t curve in 0.1 M PBS and 5.0 mM H_2_O_2_ under the applied potential of −0.5 V (Figure 6a). The CHIT/Zn-Co-S@G/GCE exhibits the strongest response current due to the combination of multiple electroactive sites in the Zn-Co-S@G nanocomposites. Then, the CHIT/Zn-Co-S@G fabricated electrode was sequentially modified with GA, anti-CA19-9, BSA, and CA19-9 to form GA/CHIT/Zn-Co-S@G/GCE, anti-CA19-9/GA/CHIT/Zn-Co-S@G/GCE, BSA/anti-CA19-9/GA/CHIT/Zn-Co-S@G/GCE, and CA19-9/BSA/anti-CA19-9/GA/CHIT/Zn-Co-S@G/GCE, respectively. It can be seen that the response current decreases gradually with the addition of non-conductive biological substances to the formation of non-conductive layers, which limit the electron transfer capacity and consequently decrease the response current. The above results from amperometry were also verified using electrochemical impedance spectroscopy (EIS) (Figure 6b), performed in 0.1 M PBS containing 5 mM [Fe(CN)_6_]^3−^ as an electrochemical probe. EIS is usually based on changes in electrical impedance of the modified electrodes. An EIS spectrum consisting of a semicircle in the high frequency region provides the value of charge transfer resistance (R_ct_) (defined as the diameter of the corresponding semicircle) that reflects the charge transfer process between the electrode/electrolyte interface. Figure 6b shows the plot for the relationship between R_ct_ and stepwise construction processes of the modified electrodes. Using the stepwise procedure for the fabrication of the immunosensor, there was a gradual increase in R_ct_, leading to a decrease in the electron-transfer rate, which is consistent with Figure 6a. It demonstrated that the electrochemical immunosensor was successfully constructed and can be used for quantification of CA19-9.

**Figure 7:**
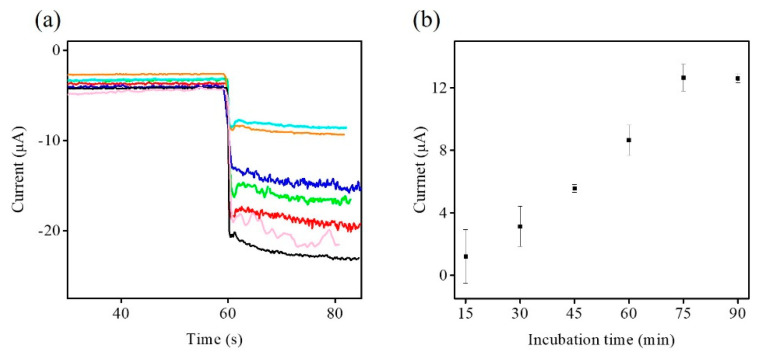
(**a**) Amperometric I-t curve of CHIT/Zn-Co-S@G/GCE in 0.1 M PBS in 5 mM H_2_O_2_ with different CA19-9 incubation times; 0 min (black line), 15 min (magenta line), 30 min (red line), 45 min (green line), 60 min (blue line), 75 min (cyan line), and 90 min (orange line) in CA19-9 at 100 U·mL^−1^. (**b**) The relationship between response current and CA19-9 incubation time in Figure 7a.

In order to achieve better performance for the following electrochemical immunosensing, the influence of the CA19-9 incubation time on response current was investigated from 0 to 90 min using CA19-9 at 100 U·mL^−1^ by the amperometric I-t curve (Figure 7a). The changes in the response current of CHIT/Zn-Co-S@G/GCE with CA19-9 incubation time is presented in the Figure 7b. It was clearly observed from Figure 7 that the response current clearly increases with increasing CA19-9 incubation time, and reaches a maximum at an incubation time of 75 min. Then, the response current tends to steady after about 75 min. Thus, an incubation time of 75 min was selected as the optimum antigen incubation time for quantification of CA19-9.

**Figure 8:**
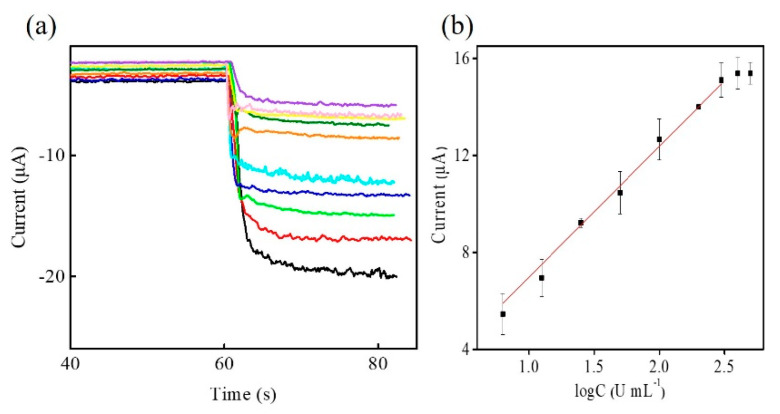
(**a**) Amperometric I-t curve of BSA/anti-CA19-9/GA/CHIT/Zn-Co-S@G/GCE at applied potential of −0.5 V in 0.1 M PBS in 5 mM H_2_O_2_ with successive addition of various CA19-9 concentrations; 0 U·mL^−1^ (black line), 6.3 U·mL^−1^ (red line), 12.5 U·mL^−1^ (green line), 25 U·mL^−1^ (blue line), 50 U·mL^−1^ (cyan line), 100 U·mL^−1^ (orange line), 200 U·mL^−1^ (olive line), 300 U·mL^−1^ (yellow line), 400 U·mL^−1^ (magenta line), and 500 U·mL^−1^ (purple line). (**b**) The linear range of BSA/anti-CA19-9/GA/CHIT/Zn-Co-S@G/GCE in Figure 8a.

After the optimization of prepared BSA/anti-CA19-9/GA/CHIT/Zn-Co-S@G/GCE, the prepared electrode was used for fabricating an efficient sensing platform for electrochemical immunosensors and enabling the label-free and sensitive immunoassay of CA19-9. Figure 8 shows a typical amperometric I-t curve of BSA/anti-CA19-9/GA/CHIT/Zn-Co-S@G/GCE in 0.1 M PBS and 5.0 mM H_2_O_2_ at applying potential −0.5 V with increasing CA19-9 concentration. The response current clearly increases with increasing CA19-9 concentration, and then the response current tends to steady after about 400 U·mL^−1^, which was saturated at a high CA19-9 concentration. Figure 8b showed the relationship between response current and CA19-9 concentration. The calibration plot exhibited a good linear relationship between response current (ΔI) and the logarithm of the CA19-9 concentration (Log C) in the range 6.3 U·mL^−1^–300 U·mL^−1^. The corresponding calibration equation is ΔI (μA) = 1.58 + 5.39 log C (U·mL^−1^), having the regression coefficient (R^2^) of 0.9974 and the limit of detection of 0.82 U·mL^−1^ based on a signal-to-noise ratio of 3 (S/N = 3). To better further evaluate the electrochemical immunosensors, the performance of our Zn-Co-S@G nanocomposite electrochemical immunosensor is compared with that of other electrochemical immunosensors for detecting the biomarker CA19-9, according to previous reports, which was better or comparable to that of the previous reports, as shown in Table 1 [1,6,26,27,28,29]. 

## 4. Conclusions

In this study, Zn-Co-S@G nanocomposites were successfully synthesized by a one-step solvothermal method. The synergistic effect between Zn-Co-S and graphene provided unique structural characteristics owing to numerous electroactive sites and short electron/ion diffusion length, illustrating the excellent electrochemical sensing properties of Zn-Co-S@G nanocomposites. It is favorable for the construction of electrochemical immunosensors and improvement of immunosensor performance. Optimization of the applied potential (−0.5 V) and the incubation time of CA19-9 (75 min) would be conducted to construct a label-free and sensitive CA19-9 biomarker sensing platform. The constructed electrochemical immunosensor exhibited a good linear relationship in the range 6.3 U·mL^−1^–300 U·mL^−1^, with a limit of detection of 0.82 U·mL^−1^. The remarkable electrochemical immunoassay of CA19-9 biomarker detection using Zn-Co-S@G nanocomposites provides a significant supporting role for early clinical cancer diagnosis and further prognosis guidance.

## Figures and Tables

**Table 1:** The performance comparison of electrochemical immunosensors for detecting the biomarker CA19-9.

Type of Materials	LOD (U·mL^−1^)	Linear Range (U·mL^−1^)	References
CeO_2_/FeOx@ mesoporous carbon	10^−5^	10^−4^–10	[1]
Polythionine-Au	0.26	6.5–520	[6]
Polythionine/sodium dodecyl sulphate	0.45	5–400	[26]
AuPt nanocalliandras	0.03	0.05–50	[27]
Carbon nanoonions/grphene oxide	0.12	0.3–100	[28]
Carbon black/polyelectrolytes	0.07	0.01–40	[29]
Zn-Co-S/graphene	0.82	6.3–300	This work

## Data Availability

Not applicable.
